# Clinical and enzymatic phenotypes in congenital hyperinsulinemic hypoglycemia due to glucokinase‐activating mutations: A report of two cases and a brief overview of the literature

**DOI:** 10.1111/jdi.13072

**Published:** 2019-06-12

**Authors:** Fan Ping, Zhixin Wang, Xinhua Xiao

**Affiliations:** ^1^ NHC Key Laboratory of Endocrinology Department of Endocrinology Peking Union Medical College Hospital Chinese Academy of Medical Science and Peking Union Beijing China; ^2^ Department of Endocrinology Beijing Jishuitan Hospital Beijing China

**Keywords:** Enzyme kinetics, Glucokinase, Hypoglycemia

## Abstract

**Aims/Introduction:**

The principal aim of this study was to investigate the clinical, genetic and functional characteristics of two cases of congenital hyperinsulinism (CHI) caused by glucokinase (GCK) mutations in young patients.

**Materials and Methods:**

Novel mutations were detected by CHI next‐generation sequencing, and the kinetic parameters and thermal stability of recombinant wild‐type and mutant glucokinase were determined *in vitro*. In addition, 18 naturally occurring GCK‐CHI mutations reported previously were also summarized.

**Results:**

A de novo mutation (M197V) was found in a 17‐year‐old male with an epilepsy history, whereas an autosomal dominant mutation (K90R) was found in a 20‐year‐old female with inherited asymptomatic hypoglycemia. Kinetic analysis showed increased enzyme activity for both mutants (RAI 4.7 for M197V and 1.6 for K90R) and enhanced thermal stability for the M197V mutant. However, of all the GCK‐CHI mutants, the increase in enzyme activity (RAI between 1.6 and 130) did not correlate strongly with the severity of hypoglycemia. The de novo group (7/19) showed distinctive phenotypes from the autosomal dominant group (12/19), such as a higher proportion of diazoxide unresponsiveness (28.6% vs 0%), a higher incidence of macrosomia (85.7% vs 40%) and a rarer incidence of adulthood onset (0% vs 25%).

**Conclusions:**

The clinical phenotypes of GCK‐CHIs were highly heterogeneous. We have identified two novel GCK‐CHI mutations in young patients and investigated their pathogenicity by enzyme kinetic analysis, which expanded the spectrum of this rare disease.

## Introduction

Glucokinase (GCK) catalyzes the first reaction of the glycolytic pathway and acts as a glucose sensor in β‐cells for adjusting the threshold of glucose‐stimulated insulin secretion^1^. GCK heterozygous inactivation mutation is one of the most commonly reported forms of maturity‐onset diabetes in young (GCK‐MODY) with the phenotypes of mild, stable fasting hyperglycemia. However, a dominant gain‐of‐function mutation of GCK remains one of the rarest forms of congenital hypoglycemia (CHI), which is a rare hereditary condition characterized by inappropriate insulin secretion. It is estimated that the incidence of GCK‐MODY is less than one in a million annually^2^, ^3^, whereas GCK‐MODY has a prevalence of one in 100^4^. Since the first case of GCK‐CHI was reported in 1998^5^, only a few variants have been identified in past 20 years. Therefore, the information about clinical characteristics and enzymatic kinetics of GCK‐CHIs is very limited, despite one reported case of a fatal mutation^6^.

In the present study, two novel GCK‐CHI mutations were identified in two young patients with unknown causes of hypoglycemia: M197V and K90R. The effects of these mutations on the enzymatic function were also explored by *in vitro* recombinant protein assays. In addition, these two mutants were compared with the 18 mutants reported in the literature so far, in an attempt to discover the characteristics of this rare disease.

## Methods

### Participants

**Figure 1:**
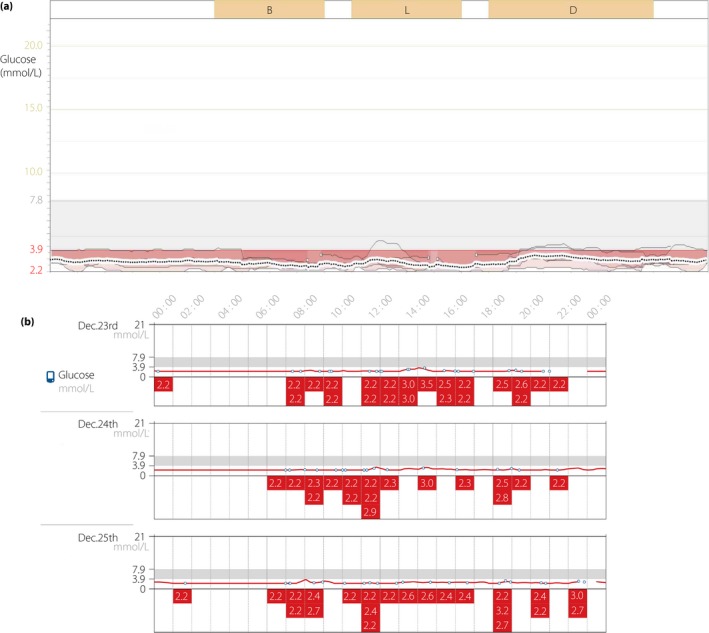
The records of continuous glucose monitoring (CGM) of case 1. (a) Conventional CGM for 6 days in the ward and (b) Flash CGM for 14 days during follow up showed persistent hypoglycemia.

Two young patients were admitted from 2013 to 2014 at Peking Union Medical College Hospital. Both patients had a suspected diagnosis of CHI for hyperinsulinemic hypoglycemia without any anatomical evidence of insulinoma. Persistent fasting hypoglycemia was stable and could be exacerbated by oral or intravenous glucose stimulation. Case 1 was a 17‐year‐old male with a large‐for‐gestational‐age birthweight of 4.75 kg at term. He had normal intelligence and development, overweight after puberty (body mass index [BMI] 24.7 kg/m^2^). At the age of 9 years, he was diagnosed with epilepsy and treated with anti‐epileptic agents. One month before admission, he suffered from excited delirium 30 min post‐lunch, during which his blood glucose (2.2 mmol/L) level was first tested. Both the conventional continuous glucose monitoring system (MiniMed Paradigm 722; Medtronic Inc., Minneapolis, MN, USA; Figure 1a) in the ward and the Flash continuous glucose monitoring system (Abbott Inc., Chicago, IL, USA; Figure 1b) during follow up showed a persistent low glucose level without obvious hypoglycemia symptoms. He was not fully responsive to diazoxide (defined as being able to fast >12 h with a glucose level of >3.9 mmol/L^7^), meanwhile, the side‐effect of diazoxide on increased fluid retention was intolerable. Case 2 was a 20‐year‐old female with birthweight of 4.6 kg, who was referred to our hospital due to asymptomatic fasting hypoglycemia (blood glucose 2.9 mmol/L) detected by a routine physical examination. She was in excellent physical and mental health, with BMI in the normal range (BMI 20.4 kg/m^2^).

### Phenotypic evaluation

A 5‐h oral glucose tolerance test with a 75‐g glucose load was undertaken in the two patients, and the fasting glucose level was determined for their family members. The serum level of creatinine, alanine aminotransferase, aspartate aminotransferase, total bilirubin, triglyceride, total cholesterol, high‐density lipoprotein cholesterol, low‐density lipoprotein cholesterol and free fatty acid were measured by an automatic biochemical analyzer (Beckman AU5800 analyzer; Beckman, Brea, CA, USA). Glycosylated hemoglobin was measured by high‐performance liquid chromatography (Varian II; Bio‐Rad, Hercules, CA, USA). The circulating lactic acid and ammonia level were determined by Siemens Dimension EXL 200. Thyroid hormones (free triiodothyronine, free thyroxine and thyroid‐stimulating hormone), sex hormones (follicle‐stimulating hormone, luteinizing hormone, estradiol and testosterone) and cortisol levels were evaluated by the ADVIA Centaur XP Immunoassay System (Siemens Healthcare GmbH, Erlangen, Germany). The urine ketone bodies and circulating insulin autoantibodies were detected when hypoglycemia occurred. The radiological evaluations including pancreatic perfusion computed tomography, pancreatic magnetic resonance imaging, endoscopic ultrasound, somatostatin receptor scintigraphy with ^99m^Tc‐hydrazino nicotinamide‐octreotide and ^68^Ga‐NOTA‐exendin‐4 positron emission tomography computed tomography^8^ were carried out to identify the possibility of insulinoma. The invasive examinations and genetic analysis were approved by the local institutional ethics committee, and written informed consent was obtained from both participants.

### Genetic analysis

DNA was extracted from the peripheral blood, using the QIAamp DNA Blood Mini Kit (Qiagen, Hilden, Germany). A customized medical panel was designed to capture approximately 11.8 Mb specific disease‐associated regions, which were used with Roche Nimblegen SeqCap EZ Choice XL Library (http://sequencing.roche.com/en/products-solutions/by-category/target-enrichment/hybridization.html). The capture sequences included the partial promoter regions, all coding exons, and 20 bp of their flanking 5′ and 3′ intronic regions. The next‐generation sequencing analysis retrieved a total of 11 genes^9^ (*ABCC8*,* KCNJ11*,* GLUD1*,* GCK*,* HADH1*,* UCP2*,* MCT1*,* HNF4A*,* HNF1A*,* HK1* and *PGM1*) for both patients. All identified variants were annotated according to the guidelines published by the Human Genome Variation Society. To assess the pathogenesis of the missense variants, different predictive software programs were used including: SIFT and Polyphen‐2 (http://sift.jcvi.org/ and http://gene.org/ and http://genetics.bwh.harvard.edu/pph/, respectively).

Sanger sequencing was carried out to confirm all the deleterious mutations and potentially pathogenic variants, and to segregate them in the families. The polymerase chain reaction products were resolved on an automated sequencer (ABI 3130xl Genetic Analyzer; Applied Biosystems, Foster City, CA, USA). The results were analyzed by SeqMan software (Madison, WI, USA) by assembling and visualizing the aligned sequences compared with the reference sequence (UCSC Genome Browser; University of California, Santa Cruz, CA, USA).

### Site‐directed mutagenesis and kinetic enzyme analysis

Preparation of recombinant proteins and enzyme kinetic analysis were carried out as previously described^10^. The Hill equation was utilized to calculate the maximum reaction rate of the enzyme (*V*_max_), glucose concentration required for GCK activity to be one‐half maximal (glucose S_0.5_) and the Hill coefficient of GCK towards glucose. The Michaelis–Menten equation was applied to calculate the Michaelis constant of the enzyme (*K*_m_) value for the second substrate (ATP‐Km, the index of affinity for adenosine triphosphate [ATP]). The rate of catalysis (*K*_cat_) was calculated by *V*_max_ / enzyme concentration. The relative activity index (RAI) of each mutant was estimated using 2.5 mmol/L intracellular ATP^11^. The kinetic parameters were analyzed by SigmaPlot software (version 12.0; Systat Software, San Jose, CA, USA)^12^. All assays were carried out in triplicate to evaluate each of the enzyme parameters, and comparisons of the continuous variables between the two groups were carried out by independent sample *t*‐tests. *P*‐values <0.05 were considered significant.

### Thermolability assays

There were two steps to determine the thermal stability of the enzyme: (i) the enzyme solutions were incubated for 30 min at different temperatures ranging from 30°C to 60°C, and analyzed at 30°C with 200 mmol/L glucose and 5 mmol/L ATP; and (ii) the enzyme solutions were incubated for different periods of time from 5 to 30 min at 52°C and then tested.

## Results

### Clinical phenotypes and GCK mutation screening

**Figure 2:**
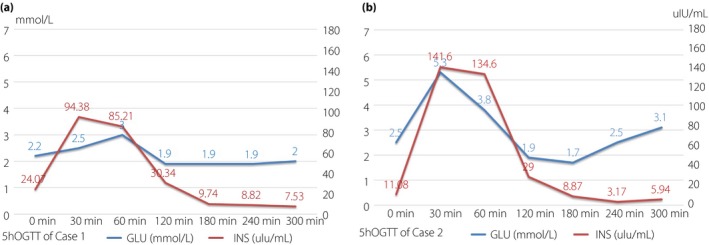
The results of 5‐h oral glucose tolerance test. (a) Case 1. (b) Case 2. Fasting hyperinsulinemic hypoglycemia was exacerbated by glucose loading. The lowest glucose level with and inappropriate high insulin level occurred at the 2–3 h after oral glucose intake. After that point, the glucose level increased slightly spontaneously, while insulin secretion was suppressed to a relatively low degree.

**Figure 3:**
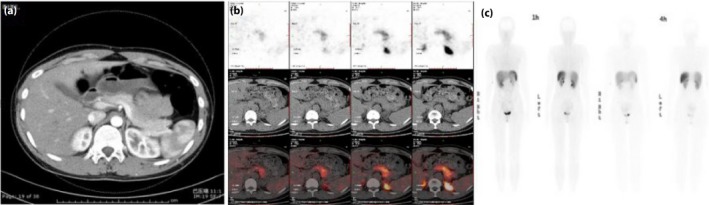
Negative results of radiological examinations. (a) Pancreatic perfusion computed tomography image of case 1. (b) 68Ga‐NOTA‐exendin‐4 positron emission tomography/computed tomography and (c) somatostatin receptor scintigraphy images with ^99m^Tc‐hydrazino nicotinamide‐octreotide of case 2.

Fasting hyperinsulinemic hypoglycemia could be exacerbated by glucose loading during oral glucose tolerance test in both patients (Figure 2). Interestingly, when the glucose level reached its lowest point, insulin secretion began to decrease by self‐regulation and then glucose concentration was increased slightly. All the radiological examinations showed no evidence of insulinoma (Figure 3). Serum alanine aminotransferase, aspartate aminotransferase, total bilirubin, ammonia, lactic acid, creatinine and lipid profiles were in the normal range, as well as the thyroid function, sex hormones and cortisol level. The urine ketone bodies and circulating insulin autoantibodies were negative during hypoglycemia episodes. Decreased glycosylated hemoglobin values (3.5% of case 1 vs 4.1% of case 2) suggested that the average glucose levels were below the normal range (Table S1).

**Figure 4:**
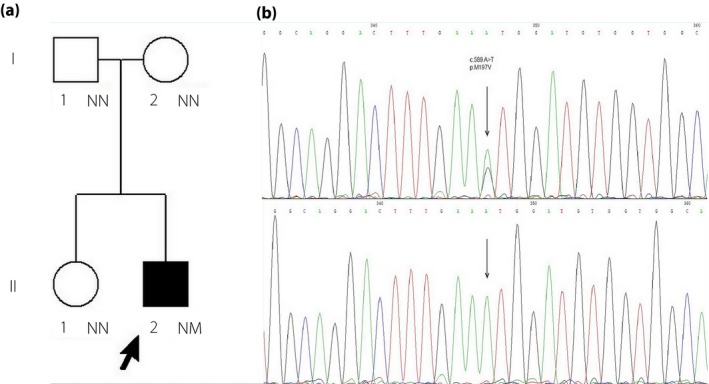
Pedigree chart and glucokinase genetic analysis of case 1. (a) Pedigree chart shows that case 1 had no family history of hypoglycemia (the arrow denotes the proband. Males are shown as squares and females are shown as circles. Filled symbols denote individuals with hypoglycemia; open symbols denote normal glycemic individuals. NN denotes no mutation, NM denotes heterozygous mutation and NT denotes not tested). Glucokinase genetic analysis result shows a de novo heterozygous mutation of case 1 in coding region: c.589 A>G, p.M197V. (b) The chromatographs of the parents and his older sister are also shown to prove the mutation is a de novo mutation.

**Figure 5:**
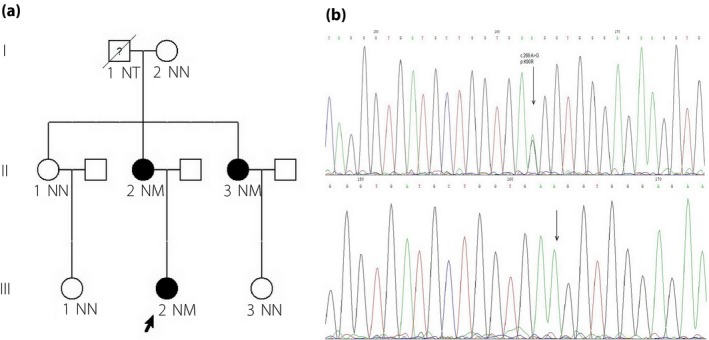
Pedigree chart and glucokinase (GCK) genetic analysis of case 2. (a) Pedigree chart shows that the mutations in this pedigree were likely inherited in an autosomal dominant pattern. The maternal grandfather (I.1) had died without genetic analysis or glucose detection. The proband's mother (II.2) and an aunt (II.3) were also found to have asymptomatic fasting hypoglycemia. The maternal grandmother (I.2) was still alive with no GCK mutation detected. (b) GCK genetic analysis of case 2 (I.1) and her living relatives: genetic analysis shows a novel heterozygous mutation in GCK coding region in case 2, her mother (II.2) and an aunt (II.3): c.269 A>G p.K90R.

As for genetic analysis, a novel GCK heterozygous mutation was identified in case 1 (c.589 A>G p.M197V; Figure 4). There were no family members of case 1 with hypoglycemia, and further genetic sequencing suggested the mutation was a de novo mutation. This was the very first case identified as naturally onset, rather than artificially constructed as previously reported^7^. A novel autosomal dominant (AD) mutation (c.269 A>G p.K90R) was identified in multiple family members of case 2 (Figure 5). The patient's mother and one of her aunts were mutant gene carriers who also had asymptomatic fasting hypoglycemia.

### *In silico* analysis of both missense mutations

The bioinformatics tools (SIFT and Polyphen2) provided inconsistent results for the pathogenicity of both M197V and K90R. The score of PolyPhen2 was 0.987 (probably damaging), and the SIFT score was 0.29 (tolerated) for M197V. Meanwhile, the PolyPhen2 score was 0.941 (probably damaging), and the SIFT score was 0.35 (tolerated) for K90R. Based on the paradoxical results of analysis *in silico*, further studies of enzyme kinetics were necessary and of great importance.

### Enzyme kinetic analyses and thermal stability assays

Protein yield and enzyme kinetic characteristics of wild‐type, M197V and K90R mutants *in vitro* are presented in Table 1. The protein yield increased in the M197V mutant, while it decreased in the K90R mutant. The *K*_cat_ and *V*_max_ were similar to the wild type in the M197V mutation, whereas they were decreased in the K90R mutation. Both M197V and K90R variants resulted in an increased affinity for glucose, greater RAI, slightly reduced Hill coefficient and increased ATP‐Km when compared with the wild type. Mutations of K90R had similar thermal stability to the wild type, whereas M197V showed a higher thermal stability after a 5‐min incubation at 52°C (61.9% vs 33.3%; Figure S1).

**Table 1:** Kinetic analysis of M197V mutant glucokinase and K90R mutant glucokinase

GCK	Yield (mg/L)	S_0.5_ (mmol/L)	Hill coefficient	ATP‐Km (mmol/L)	*V*_max_ (U/mg)	*K*_cat_ (s^−1^)	RAI
Wild type	85.8 ± 8.7	7.63 ± 0.21	1.42 ± 0.06	0.3 ± 0.01	9.83 ± 1.01	20.9 ± 2.1	1.0
M197V	108.2 ± 4.1*	2.57 ± 0.12*	1.22 ± 0.03*	0.33 ± 0.01*	8.13 ± 0.55	17.3 ± 1.2	4.7 ± 0.6*
K90R	70.4 ± 3.5*	4.8 ± 0.26*	1.23 ± 0.02*	0.38 ± 0.02*	6.33 ± 1.06	13.5 ± 2.3*	1.6 ± 0.2*

*P < 0.05 vs wild‐type values. Maximal glucose concentration was 100 mmol/L by the kinetic analysis of the enzyme. Expression and purification of the wild‐type GCK and M197V and K90R mutants were repeated three times each, and all functional assays were performed in triplicate. ATP‐Km, adenosine triphosphate affinity for the second substrate adenosine triphosphate concentration at half‐maximal reaction rate when glucose levels were saturated; GCK, glucokinase; *K*_cat_, the catalytic activity; RAI, the relative activity index of glucokinase (when ATP‐Mm was 0.3 mmol/L); S_0.5_, glucose concentration at half‐maximal reaction rate when adenosine triphosphate concentrations were 5 mmol/L; *V*_max_, the maximum reaction rate of the enzyme.

### Brief overview of naturally occurring GCK‐CHIs

To date, just 20 cases of naturally occurring GCK‐CHI mutations were reported, including the two variants in the present study (Table 2), sorting by amino acid sequences as S64Y^3^, T65I^13^, G68V^14^, K90R (case 2 of this study), V91L^15^, W99R^13^, W99L^7^, W99C^16^, T103S^11^, N180D^17^, M197I^7^, M197T^18^, M197V (case 1 of this study), Y214C^6^, V389L^11^, ^19^, E442K^20^, V452L^21^, ^22^, ins454A^7^, V455M^5^ and A456V^12^. Of the 20 GCK‐CHI mutations, the missense mutations account for 95% (19/20), and the remaining mutation was an insertional mutation (5%, 1/20). Except for one case with unidentified family history, the other 19 mutations can be divided into two groups based on the inheritance pattern: approximately one‐third of mutations (36.8%, 7/19) were in the de novo group, whereas the rest were in the AD group (63.2%, 12/19). The clinical manifestations caused by GCK‐CHI were highly heterogeneous on different genotypes. More than half of the probands (10/18) were born with macrosomia, and the prevalence of this trait was higher in the de novo group than the AD group (85.7% vs 40%). The onset age varied from birth to 44 years. However, 15 out of 20 probands developed hypoglycemia before the age of 10 years. Unfortunately, the average age of confirmatory diagnosis was at the age of 16 years, suggesting that many patients had been misdiagnosed for a long time. Among the four variants that presented adult onset, three were identified as AD inheritance.

**Table 2:** Clinical and functional characterizations of naturally occurring congenital hyperinsulinism caused by glucokinase mutations

	Nucleotide/protein position	Proband's age/sex (onset age)	Inheritance patterns	BW (kg)	BG (mmol/L)/INS (uU/mL)†	Diazoxide response	Kinetic parameters of mutants				
							S_0.5_	ATP‐Km	Kcat	h	RAI
1	c.191C>A p.S64Y^3^	17 years/M (1 day)	De novo	4.3	2.0/12	Yes	1.5	↑	↓	↓	22
2	c.194C>T p.T65I^13^	15 years/M (neonate)	AD	3.1	2.2/15.5	Yes	1.7	↑	↓	↓	9.8
3	c.203G>T p.G68V^14^	7 years/F (7 months)	AD	3.7	1.6/.7.6	Yes	1.9	↓	↑	↓	16
4	c.269A>G p.K90R	20 years/F (20 years)	AD	4.6	2.5/11.1	NA	4.8	↑	↓	↓	1.6
5	c.271C>G p.V91L^15^, ^16^	2 years/F (1 day)^16^	AD	Macrosomia	1.7/20	Yes	1.7	↔	↔	↓	24
6	c.295T>A p.W99R^13^	10 months/M (1 day)	AD	3.1	2.4/5.2	Partial	4.9	↑	↑	↓	4.1
7	c.296G>T p.W99L^7^	6 years/M (6 y)	De novo	3.2	2.7/NA	Partial	0.9	↔	↑	↓	8.9
8	c.297G>T p.W99C^16^	25 years/F (25 years)	NA	2.4	2.9/13.5	Yes	3.5	↓	↑	↓	11.6
9	c.308C>G p.T103S^11^	22 years/F (15 years)	AD	3.2	2.8/6.0	Yes	3.3	↔	↓	↔	8.4
10	c.538A > G p.N180D^17^	25 years/F (childhood)	AD	NA	2.1/30	Partial	NA	NA	NA	NA	NA
11	c.589 A>G p.M197V	17 years/M (9 years)	De novo	4.8	2.2/24.1	Partial	2.6	↑	↓	↓	4.7
12	c.591G>T p.M197I^7^	16 years/M (1 h)	De novo	4.9	2.2/NA	Yes	2.6	↑	↓	↓	3.1
13	c.590T>C p.M197T^7^, ^18^	56 years/F (44 years)	AD	NA	2.6/3.0	NA	4.1	↑	↓	↓	2.9
14	c.641A>G p.Y214C^6^	29 years‡/F (1 day)	De novo	4.4	2/2.9	No	1.2	↑	↑	↓	130
15	c.1165C>G p.V389L^11^, ^19^	3.6 years/M (2 years)	AD	5.4	2.9/6.6	Partial	3.5	↑	↑	↔	6.0
16	c.1324G>A p.E442K^20^	6 years/F (1 day)	AD	2.8	1.5/NA	Yes	4.4	↑	↓	↓	3.3
17	c.1354G>C p.V452L^21^	3 years/M (1 day)	De novo	5.9	2.5/2.5	Yes	2.6	↑	↓	↓	10.8
18	c.1361‐364 insCGG p.ins454A^7^	17 years/M (1 h)	De novo	4.8	2.2/11.5	NO	1.1	↓	↓	↓	26
19	c.1363C>A p.V455M^5^, ^7^	31 years/M (31 years)	AD	4.3	2.5/29	Yes	2.9	↔	↔	↔	5.2
20	c.1367C>T p.A456V^12^	14 years/M (1 day)	AD	3.8	3.1/37	Yes	2.5	↓	↑	↓	37.9

^†^BG (mmol/L)/INS (uU/mL, 1 uU/mL = 6 pmol/L) denotes simultaneous blood glucose (BG) and insulin (INS) level during a hypoglycemia episode. ^‡^At 29 years‐of‐age, the proband was dead. Fully responsive to diazoxide was defined as being able to fast >12 h with plasma glucose of >3.9 mmol/L. ↓ decreased, ↑ increased, ↔ unchanged; AD, autosomal dominant; ATP‐Km, adenosine triphosphate affinity for the second substrate adenosine triphosphate concentration at half‐maximal reaction rate when glucose levels were saturated; BW, birthweight; F, female; h, Hill coefficient; *K*_cat_, the catalytic activity; M, male; NA, not available; RAI, relative activity index.

During the hypoglycemia episode, the average value of blood glucose was 2.3 mmol/L (range 1.5–3.1 mmol/L) and for insulin level was 14.0 uU/mL (range 2.5–37 uU/mL). However, the insulin levels in probands caused by three variants (M197T, V452L and Y214C) did not exceed 3 uU/mL (18 pmol/L) during hypoglycemia episodes, which did not conform to the typical criteria of endogenous hyperinsulinemic hypoglycemia^23^. The severity of hypoglycemia symptoms ranged from asymptomatic to epilepsy, coma and even death^6^. More than half of the GCK‐CHIs (61.1%, 11/18) were fully responsive to diazoxide, whereas five out of 18 (27.8%) were partially responsive and two out of 18 (11.1%) were unresponsive. Interestingly, the percentage of diazoxide unresponsiveness in the de novo group (28.6%, 2/7) appeared to be higher than that in the AD group (0%, 0/11).

Enzyme function was verified in 19 of 20 GCK‐CHI mutants, and is briefly summarized in Table 2. The change of catalytic activity (*K*_cat_) of mutants was inconsistent: 52.6% (10/19) decreased, 36.8% (7/19) increased and 10.5% (2/19) remained unchanged. This inconsistency was also seen in the parameter reflecting the affinity for ATP binding (ATP‐Km): 68.4% (11/19) increased, 21.1% (4/19) decreased and 21.1% (4/19) remained unchanged. The index of cooperativity (Hill coefficient) for glucose reduced in most GCK‐CHIs (84.2%, 16/19), but remained unchanged in 15.7% (3/19) of GCK‐CHIs. Interestingly, all the variants with an unchanged Hill coefficient were in the AD group. All the GCK‐CHIs had lower S_0.5_ (0.9–4.9 mmol/L) *in vitro*, which was in contrast with the wild‐type S_0.5_ (7.5–10 mmol/L), and glucose S_0.5_ indicates the glucose concentration required for GCK activity to be one‐half maximal. These vital kinetic changes resulted in increased enzymatic RAI from 1.6 to 130. Although the Y214C variant with RAI of 130 caused the most severe hypoglycemia from birth and early death^6^, the correlations between varying degrees of elevated RAI and the severity of clinical phenotypes remained elusive. For instance, M197I^7^with RAI 3.1 resulted in hypoglycemia soon after birth, whereas T103S^11^with RAI 8.4 caused hypoglycemia at 15 years‐of‐age.

## Discussion

To date, >800 types of GCK mutations have been reported, and the majority were focused on phenotypes of hyperglycemia. GCK‐MODY caused by heterozygous inactivating mutations is a mild hyperglycemia condition, which generally does not require medical interventions and rarely leads to diabetic complications^24^. At the same time, little is known about the phenotype characteristics of GCK‐CHIs. In the present study, two cases of congenital hyperinsulinism due to novel GCK activating mutations were investigated, and enzyme kinetic data were analyzed *in vitro*. In addition, to explore the characteristics of patients with this rare disease, the present study provided a brief overview of all the naturally occurring GCK‐CHIs.

The probands of two activating mutations described in this report were both born with macrosomia, but the rest of the clinical characteristics were highly heterogeneous, such as different body shapes, symptoms of hypoglycemia and inheritance patterns. Our *in vitro* studies also showed increased enzyme activities for both mutants, and increased thermal stability for the M197V mutant. A recent report suggests two mechanisms of GCK activation among the gain‐of‐function mutations: α‐type activation results from a shift in the conformational ensemble of unliganded GCK toward a state resembling the glucose‐bound conformation, whereas β‐type activation is attributable to an accelerated rate of production release^25^. It was reported that V91 was located in the allosteric activator region^26^, and as a close neighbor, K90R is likely to be directly activated by structural changes and appears to be α‐type. In contrast, M197 is 19 Å away from the GCK activator site and therefore unlikely to interfere with activator binding^7^. *In vitro* studies have shown that the activating mutation of this site increased proteolytic susceptibility without substantially altering the affinity toward glucose^27^, and therefore, is more likely to be the β‐type. Two naturally occurring mutations, M197I and M197T^7^, ^18^, have been described in hypoglycemia cases, and their pathogenicity was confirmed by *in vitro* studies. An artificial M197V mutant has been constructed previously^7^, but the present study identified this mutation in a natural case for the first time, further supporting the significance of this locus. Interestingly, the clinical phenotypes in M197V were more severe than those in K90R, which might be partially explained by a greater difference in biochemical property between methionine (M) and valine (V) compared with a conservative change from lysine (K) and arginine (R).

In addition to the two novel cases, an extensive literature research was carried out and summarized. Among 18 naturally occurring GCK‐CHIs, the de novo type seemed to present more consequential clinical manifestations, such as a higher proportion of diazoxide unresponsiveness, a higher incidence of macrosomia and a rarer incidence of adulthood onset. It can only be speculated that if the fetus mutation is a de novo mutation, the chance of exposure to intrauterine hyperinsulinemia will be significantly increased, which leads to a higher risk of macrosomia. One grave case was a Finnish woman with a Y214C mutation who showed severe hypoglycemia from birth, which caused irreversible brain damage and death at the age of 29 years^6^. In contrast, the phenotypes of hypoglycemia in the AD group were relatively mild, and in many of the cases, their hypoglycemia disorders escaped recognition in childhood and even adult life, such as the K90R mutant in the present study. The AD group also showed higher heterogeneity in the clinical course than the de novo group. For example, most living carriers of the V455M mutation developed diabetes later in life, suggesting an eventual β‐cell exhaustion^5^, ^28^. However, β‐cell exhaustion might not occur in all AD patients, as a carrier of the V389L mutation was reported to show persistent hypoglycemia when aged in his 90s^19^.

The present two cases, together with previous reports, suggest that the severity of clinical phenotypes might not be completely parallel to the degree of elevated RAI *in vitro*. The glucose modulation of GCK *in vivo* is much more complex than that *in vitro*, which is attributed to the regulation by the liver and the adipose tissue. It has been confirmed that the change in the interaction of GCK mutants with glucokinase regulatory protein, a hepatocyte‐specific inhibitor, can also affect the GCK activity in the liver. In addition, excessive adipose tissue might upregulate pancreatic GCK expression, as shown by the striking contrast in the clinical features of family members of A456V mutated family members with different BMIs^12^. Therefore, the pathogenic mechanisms of GCK mutation might not be limited to the level of altered enzyme kinetics, and the assays carried out *in vitro* might not be comprehensive enough in this respect. *In vivo* assays, such as animal knockout models, might be considered to determine the functional alteration of GCK when *in vitro* results are inconclusive.

In summary, we have identified two novel GCK‐CHI mutations and investigated their pathogenicity by enzyme kinetic analysis. Furthermore, a brief literature review showed that GCK‐CHIs have a broad spectrum of clinical course, and there is an apparent lack of a strong correlation between enzymatic assay and clinical symptoms. In addition, de novo and AD mutants might show different phenotypes, which warrants further investigation.

## Disclosure

The authors declare no conflict of interest.

## Supporting information


**Table S1**| Biochemical examination results of the two cases in the present study.

**Figure S1**| Thermal stability of each mutant and wild type of glucokinase (GCK). (a) The enzyme solutions were incubated for 30 min at different temperatures ranging from 30°C to 60°C, and analyzed at 30°C with 200 mmol/L glucose and 5 mmol/L adenosine triphosphate. The enzyme solutions were incubated for different periods of time from 5 to 30 min at 52°C. (b) Mutants of K90R and M197V showed thermal stability similar to that of the wild type, except that after a 5‐min incubation at temperature of 52°C the relative activity of M197V was higher than the wild type.
