# Supplementary figures and images for: Clinical and enzymatic phenotypes in congenital hyperinsulinemic hypoglycemia due to glucokinase‐activating mutations: A report of two cases and a brief overview of the literature
Source: J Diabetes Investig. 2019 Jun 12;10(6):1454–62. doi: 10.1111/jdi.13072 (PMC6825936; doi:10.1111/jdi.13072)

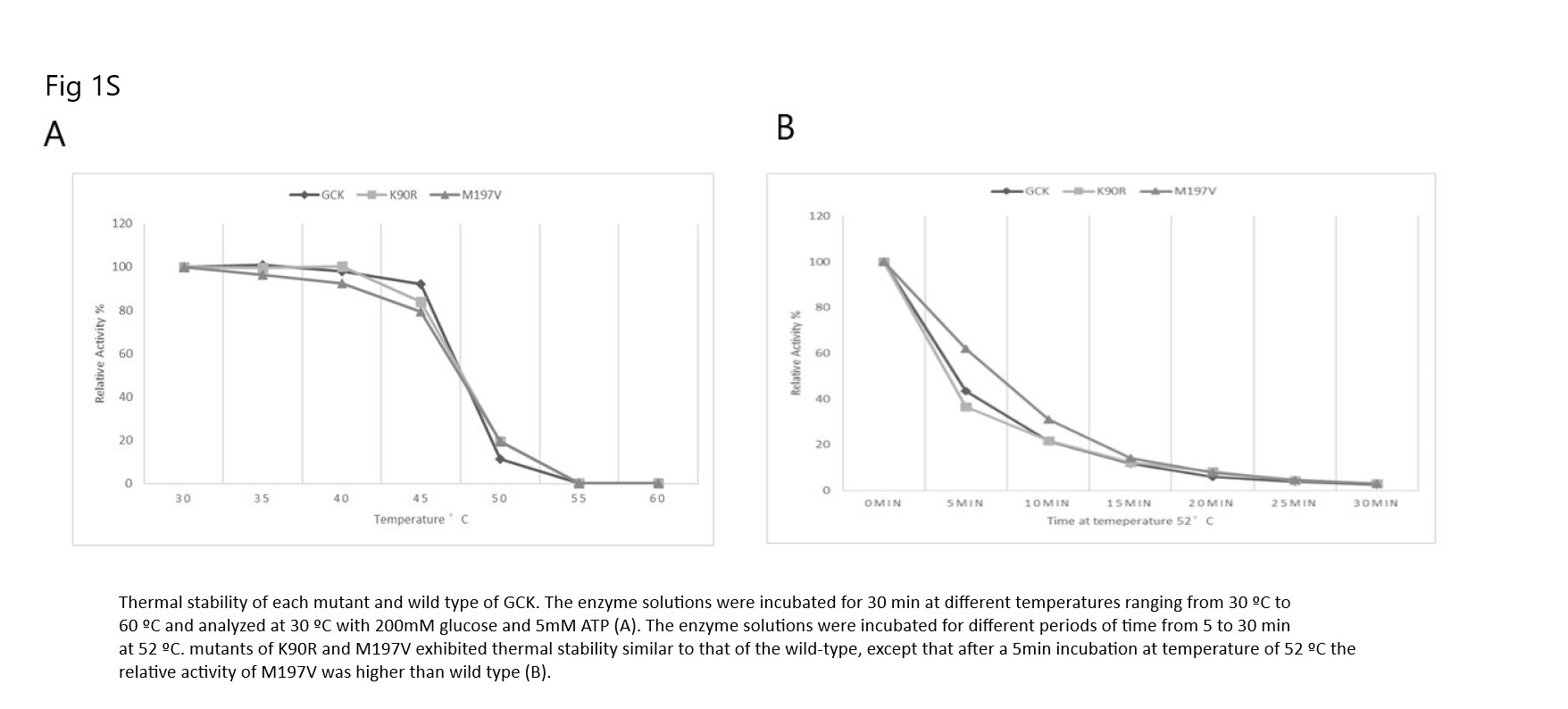

Supplement: Supplementary file 1 — Table S1| Biochemical examination results of the two cases in the present study. [file JDI-10-1454-s001.jpg]
