# Supplementary material for: Clinical and enzymatic phenotypes in congenital hyperinsulinemic hypoglycemia due to glucokinase‐activating mutations: A report of two cases and a brief overview of the literature
Source: J Diabetes Investig. 2019 Jun 12;10(6):1454–62. doi: 10.1111/jdi.13072 (PMC6825936; doi:10.1111/jdi.13072)
Supplement: Supplementary file 2 — Figure S1| Thermal stability of each mutant and wild type of glucokinase (GCK). (a) The enzyme solutions were incubated for 30 min at different temperatures ranging from 30°C to 60°C, and analyzed at 30°C with 200 mmol/L glucose and 5 mmol/L adenosine triphosphate. The enzyme solutions were incubated for different periods of time from 5 to 30 min at 52°C. (b) Mutants of K90R and M197V showed thermal stability similar to that of the wild type, except that after a 5‐min incubation at temperature of 52°C the relative activity of M197V was higher than the wild type. [file JDI-10-1454-s002.docx]

Table S1: Biochemical examination results of two cases of our study

|  | Case 1 | Case 2 |
| --- | --- | --- |
| Serum creatinine µmol/L (45–84) | 70 | 70 |
| Urea mmol/L (2.78-7.14) | 5.11 | 7.25 |
| ALT U/L (9–50) | 22 | 9 |
| AST U/L (15–40) | 23 | 16 |
| Total bilirubin µmol/L (5.1-22.2) | 8.7 | 7.4 |
| Free T3 pg/mL (1.8–4.1) | 3.36 | 2.0 |
| Free T4 ng/dL (0.81–1.89) | 1.17 | 0.87 |
| TSH µIU/mL (0.38–4.34) | 1.94 | 0.71 |
| Cortisol µg/dL (4–22.3) | 16.06 | 13.5 |
| ACTH pg/mL (0–46) | 31.5 | 21.6 |
| IGF-1 ng/mL (141–483) | 349 | 275 |
| FSH mIU/mL | 4.06 | 4.3 |
| LH mIU/mL | 7.37 | 9.56 |
| Estradiol pg/mL | 58 | 185.0 |
| Testosterone ng/dL | 451.0 | 9.0 |
| LA mM (0.5–1.6) | 1.58 | 0.31 |
| Ammonia µM (11–35) | 29 | 28 |
| FFA µM (129–769) | 156 | 144 |
| TG mM (0.45–1.7) | 0.81 | 0.4 |
| TC mM (2.85–5.7) | 3.52 | 4.2 |
| HDL-C mM (0.93–1.81) | 1.04 | 1.6 |
| LDL-C mM (2.07–3.63) | 2.22 | 2.05 |
| IAA* | NEG | NEG |
| HbA1c % | 3.5 | 4.1 |
| Urine ketone body* | NEG | NEG |

*Blood and urine samples were collected simultaneously at hypoglycemia episode; ALT, alanine aminotransferase; AST, aspartate aminotransferase; T3, triiodothyronine; T4, thyroxine; ACTH, adrenocorticotropic hormone; IGF-1, insulin-like growth factor-1; FSH, Follicle stimulating hormone; LH, luteinizing hormone; LA, lactic acid; FFA, free fatty acid; TG, triglyceride; TC, total cholesterol; LDL-C, low-density lipoprotein cholesterol; IAA, insulin autoantibody
